# Interobserver Reliability of Four Diagnostic Methods Using Traditional Korean Medicine for Stroke Patients

**DOI:** 10.1155/2014/465471

**Published:** 2014-12-09

**Authors:** Ju Ah Lee, Mi Mi Ko, Byoung-Kab Kang, Terje Alraek, Stephen Birch, Myeong Soo Lee

**Affiliations:** ^1^Medical Research Division, Korea Institute of Oriental Medicine, 1672 Yuseongdae-ro, Yuseong-gu, Daejeon 305-811, Republic of Korea; ^2^Norwegian School of Health Sciences, Institute of Acupuncture, Kristiania University College, Prinsensgate 7-9, 0152 Oslo, Norway; ^3^Department of Community Medicine, Faculty of Health Sciences, UiT The Arctic University of Norway, National Research Center in Complementary and Alternative Medicine (NAFKAM), Tromsø, Norway

## Abstract

*Objective*. The aim of this study is to evaluate the consistency of pattern identification (PI), a set of diagnostic indicators used by traditional Korean medicine (TKM) clinicians. *Methods*. A total of 168 stroke patients who were admitted into oriental medical university hospitals from June 2012 through January 2013 were included in the study. Using the PI indicators, each patient was independently diagnosed by two experts from the same department. Interobserver consistency was assessed by simple percentage agreement as well as by kappa and AC_1_ statistics. *Results*. Interobserver agreement on the PI indicators (for all patients) was generally high: pulse diagnosis signs (AC_1_ = 0.66–0.89); inspection signs (AC_1_ = 0.66–0.95); listening/smelling signs (AC_1_ = 0.67–0.88); and inquiry signs (AC_1_ = 0.62–0.94). *Conclusion*. In four examinations, there was moderate agreement between the clinicians on the PI indicators. To improve clinician consistency (e.g., in the diagnostic criteria used), it is necessary to analyze the reasons for inconsistency and to improve clinician training.

## 1. Introduction

In traditional Korean medicine (TKM) and traditional Chinese medicine (TCM), the diagnostic process is called pattern identification (PI) or syndrome differentiation [1]. TKM or TCM clinicians use the PI system to diagnose the cause, nature, and location of the illness as well as the patient's physical condition and the patient's treatment; they also determine the appropriate treatment (e.g., acupuncture, herbal medicine, and moxibustion) [2]. Therefore, the PI system plays an important role in TCM and TKM. The PI system is a synthetic and analytical process that analyzes information obtained from four examinations.

The term “four examinations” is a general term that includes visual inspection, listening and smelling, inquiry, and pulse diagnosis [1]. To successfully perform PI, an objective and precise process using the four examinations is essential.

However, the clinical competence of this process is determined by the experience and the knowledge of the clinicians. Several environmental factors, such as the differences between light sources and brightness levels, can significantly influence the visual inspection. Additionally, subjective factors, such as the patient's emotion and the clinician's interrogatory approach or technical skills, can significantly influence the examination. Pulse diagnosis is also determined by the clinician's experience and knowledge [3]. Further, many experiences in the traditional four examinations have not been scientifically or quantitatively verified. Therefore, additional studies are required to improve the reproducibility and objectivity of the TCM and TKM diagnostic processes.

Interobserver reproducibility is regarded as one of the foundations of high quality research design [4]. Many common clinical symptoms and signs fail to overcome the lack of reliability limitations when they are subjected to an interobserver study [5].

Previous reports have described the interobserver reliability of pulse diagnosis, tongue diagnosis, and PI for stroke patients [5–9]. However, the actual diagnoses are conducted by pooling information from the four diagnostic methods [9]. Therefore, in this study, we investigated the reliability of the TKM four examinations with stroke patients by evaluating the interobserver reliability regarding how these indicators demonstrated the signs or symptoms that were observed by TKM clinicians.

## 2. Methods

### 2.1. Participants

**Figure 1:**
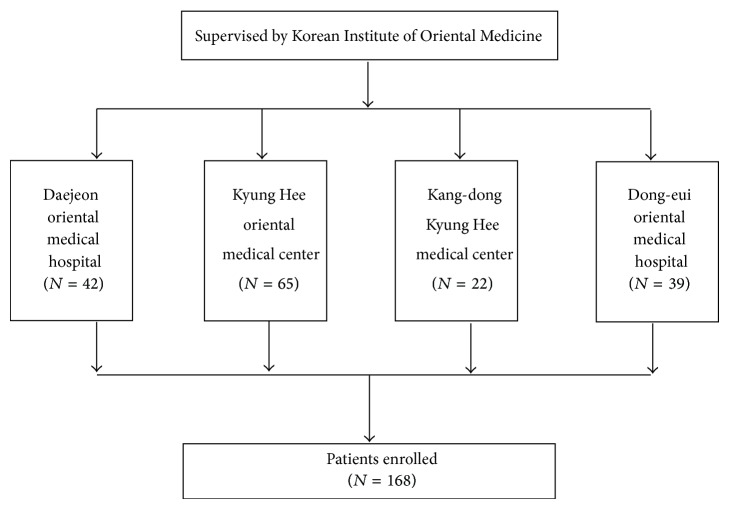
Flow diagram of patients enrolled in the study.

Data for this analysis were collected from a multicenter study of the standardization and objectification of pattern identification in traditional Korean medicine for stroke (SOPI-Stroke) [6, 10, 11]. Stroke patients were admitted between June 2012 and January 2013 to the following oriental medical university hospitals: Kyung Hee Oriental Medical Center (Seoul), Kang dong Kyung Hee Medical Center (Seoul), Daejeon Oriental Medical Hospital (Daejeon), and Dong-eui Oriental Medical Hospital (Pusan) (Figure 1). All patients provided informed consent, according to the procedures that were approved by the institutional review boards (IRBs) at the participating institutions. The following inclusion criteria were applied. The participants had to be enrolled in the study as stroke patients within 30 days of the onset of their symptoms, as confirmed by imaging diagnosis, such as computerized tomography (CT) or magnetic resonance imaging (MRI). Traumatic stroke patients, such as those with subarachnoid, subdural, or epidural hemorrhage, were excluded from the study. The present study was approved by the IRB of the Korean Institute of Oriental Medicine (KIOM) and by each of the oriental medical university hospitals.

In particular, the clinicians had to measure stroke PI of each patient following the fire-heat pattern, the phlegm-dampness pattern, the qi deficiency pattern, and the yin deficiency pattern, as suggested by the KIOM [5].

### 2.2. Data Processing and Analysis

All patients were examined by two experts (from the same TKM department) who were well trained in standard operation procedures (SOPs). The patients were subjected to the following diagnoses: pulse diagnosis (pulse location: floating or sunken, pulse rate: slow or rapid, pulse force: strong or weak, and pulse shape: slippery, fine, or surging); inspection (tongue: color, fur color, fur quality, special tongue appearance, facial complexion, abnormal eye appearance, body type, mouth, and vigor); listening and smelling (vocal sound energy and sputum, tongue and mouth, and particularly fetid mouth odor); and inquiry (headache, tongue and mouth: dry mouth and thirst in the mouth, temperature, chest, sleep, sweating, urine, and vigor). The examination parameters were extracted from portions of a case report form (CRF) for the PI for stroke, which was developed by an expert committee organized by the KIOM. These assessments were individually and independently conducted without discussion among the clinicians. The descriptions for grading the severity of each variable were scored as follows: 1 = very significant; 2 = significant; and 3 = not significant. Interobserver reliability was measured using the simple percentage agreement, Cohen's kappa coefficient, and Gwet's AC_1_ statistic [12] as well as the corresponding confidence intervals (CI). For most purposes,* kappa* values ≤0.40 represent poor agreement, values between 0.40 and 0.75 represent moderate-to-good agreement, and values ≥0.75 indicate excellent agreement [13]. The AC_1_ statistic is not vulnerable to the well-known paradoxes that make kappa appear to be ineffective [12, 14, 15]. Data were statistically analyzed using SAS software, version 9.1.3 (SAS Institute Inc., Cary, NC, USA).

## 3. Results

The general characteristics of the study subjects are shown in Table 1. The interobserver reliability results regarding pulse diagnosis domain for all subjects (*n* = 168) are shown in Table 2. The kappa value measures of agreement for the two experts ranged from “poor” (*κ* = 0.37) to “moderate” (*κ* = 0.61). The AC_1_ measures of agreement for the two experts were generally high for pulse diagnosis domain and ranged from 0.66 to 0.89.

The interobserver reliability results regarding visual inspection domain for all subjects are shown in Table 3. The kappa value measures of agreement for the two experts ranged from “poor” (*κ* = 0.26) to “moderate” (*κ* = 0.84). The AC_1_ measures of agreement for the two experts were generally high for the inspection signs and ranged from 0.66 to 0.95. The interobserver agreement was nearly perfect for several signs (e.g., mirror tongue and aphtha and sores of tongue/mouth indicators, AC_1_ = 0.95 and AC_1_ = 0.91).

The interobserver reliability results regarding the listening and smelling domain for all subjects are shown in Table 4. The kappa value measures of agreement for the two experts were “moderate” (*κ* = 0.60). The AC_1_ measures of agreement for the two experts were generally high for the observation signs and ranged from 0.67 to 0.88.

The interobserver reliability results regarding the inquiry domain for all subjects are shown in Table 5. The kappa value measures of agreement for the two experts ranged from “poor” (*κ* = 0.27) to “moderate” (*κ* = 0.76). The AC_1_ measures of agreement for the two experts were generally high for the inquiry signs and ranged from 0.62 to 0.94. Agreement, as assessed by the kappa values, was considerably lower than the AC_1 _values in the majority of cases.

## 4. Discussion

Recently, several studies have investigated the importance of education in the PI process [16, 17]. Additionally, several studies have focused on the reliability of a clinician's decision regarding PI [4, 18–20]. However, PI is achieved by comprehensively analyzing the signs or symptoms of the four examinations and it refers to a comprehensive consideration of the data obtained from these examinations [1]. Therefore, it is necessary to check the reliability among clinicians for each sign or symptom that is used to diagnose PI. Very few studies reported about importance of diagnostic variables in the four examinations [21–23]. This study aimed to use AC_1 _and kappa statistics to assess the interobserver reliability of the signs or symptoms of PI in stroke patients. Finally, we aimed to improve the objectivity and reproducibility of the PI decisions among clinicians. For convenience, all signs and symptoms are referred to as indicators.

Palpation means touching and pressing the body surface using the fingers to diagnose the pulse diagnosis [1]. Regarding interobserver agreement for pulse diagnosis among all subjects, we found that one item (fine pulse) had a poor kappa value; however, 8 items had moderate-to-good values. In particular, fine pulse had a poor value compared to other items of kappa value; but it did not have a poor value for the percentage agreement and AC_1_. We realized that many clinicians checked “3 = not significant” because of difficulties in detecting low-frequency appearance. Therefore, contrary to the kappa value, in the percentage agreement and AC_1_, there were high values (93.29%, 0.93), respectively. Pulse diagnosis has many limitations because the clinical skill of four diagnoses depends on the clinician's experience and knowledge; moreover, environmental factors have a considerable influence on the clinician's willingness. However, the results in this study showed that pulse diagnosis has good agreement.

Visual inspection means observing the patient's mental state, facial expression, complexion, and physical condition as well as the condition of the tongue [1]. Regarding interobserver inspection agreement, we found that two items (dry fur and teeth marked tongue) had poor kappa values. However, the other items had moderate-to-good values. Tongue diagnosis is the inspection of the size, shape, color, and moisture of the tongue proper and its coating [1]. Several studies have emphasized the interobserver reliability among clinicians regarding tongue diagnosis [24, 25]. Inspection, including tongue diagnosis, has unavoidable limitations because the clinical skills of observation and diagnosis depend on the clinician's experience and knowledge, and environmental factors can influence whether the clinician can obtain diagnostic results from the patient's body. Therefore, to improve the consistency of inspection, it is necessary to standardize the process and inspection skills.

The listening and smelling diagnosis constitutes one of the four examinations. Listening specifically focuses on listening to the patient's voice, breathing sounds, cough, vomiting, and so forth. Smelling is the smell from a patient's body or mouth [1]. Regarding interobserver agreement of listening and smelling diagnosis among all subjects, we found that 3 items had moderate-to-good values. Numerous studies have scored the listening and smelling diagnosis low compared with the other examinations. Therefore, additional studies of the listening and smelling diagnosis are warranted.

Inquiry, which is one of the four diagnostic examinations, is used to gain information concerning diagnosis by asking the patient about the complaint and the history of the illness [1]. We found that one inquiry item (an unpleasant sensation with an urge to vomit) had a poor kappa value.

Although there were no large differences among the diagnoses, pulse diagnosis had a low AC_1_ value. However, the results are better than those reported in a previous study [7, 8]. It is thought that clinicians have been trained in SOPs many times for this diagnosis.

In this study, simple percentage agreements and kappa value and AC_1_ statistics were used to evaluate the interobserver reliability of TKM clinicians for PI indicators in stroke patients. When investigating observer agreement, clinicians have long used kappa values and other chance-adjusted measures, with a commonly used scale for interpreting kappa [26]. However, the appropriateness of kappa value as a measure of agreement has recently been debated [14, 15]. According to published research, the AC_1_ statistic has been suggested to adjust for chance agreement [12, 27].

In TKM and TCM, the primary problem is the reproducibility of the diagnosis and the lack of objectivity. To solve these problems, interobserver reliability of PI should be increased. Thus, the interobserver reliability of indicators should be increased. To overcome these issues in the larger stroke study, the researchers regularly conducted SOPs training, and shortcomings were identified. Therefore, it is necessary that diagnostic indicators should be standardized to improve agreement among clinicians. As a result of these efforts, standardization of the TCM and TKM diagnosis will likely be achieved in the near future. In this study, there are a few limitations. First, only two raters were included in this study. Second, this study project focused on certain kinds of signs and symptoms relevant for stroke. Therefore, the study is limited on the generalizability of findings to the general field of TCM/TKM.

## Figures and Tables

**Table 1:** Demographic parameters of study subjects.

Characteristics	
*N*	168
Sex (M/F)	75/93
Age (mean ± SD)	68.89 ± 10.92
Weight (kg) (mean ± SD)	61.02 ± 11.07
Height (cm) (mean ± SD)	161.15 ± 9.04
BMI (mean ± SD)	23.41 ± 3.26
WHR (mean ± SD)	0.93 ± 0.07
WC (cm) (mean ± SD)	85.76 ± 10.08
HC (cm) (mean ± SD)	92.50 ± 7.28
TOAST classification	
LAA	46
CE	6
SVO	113
SOE	1
Others	2
Hypertension (yes/no)	103/65
Hyperlipidemia (yes/no)	25/143
DM (yes/no)	47/121
Smoking (none/stop/active)	109/18/39
Drinking (none/stop/active)	104/8/55

BMI: body mass index. WHR: waist hip ratio. WC: waist circumference. HC: hip circumference. TOAST: trial of ORG 10172 in acute stroke Treatment. LAA: large-artery atherosclerosis. CE: cardioembolism. SVO: small-vessel occlusion. SOE: stroke of other etiology. SUE: stroke of undetermined etiology. DM: diabetes mellitus.

**Table 2:** Agreement between raters in total subjects (diagnosis by palpation; pulse diagnosis).

Variables	% Agreement	Kappa (K)	CI of K	AC_1_	CI of AC_1_
Pulse location:					
Floating	88.02	0.53	(0.35, 0.71)	0.84	(0.77, 0.91)
Sunken	85.02	0.56	(0.41, 0.72)	0.77	(0.68, 0.87)
Pulse rate:					
Slow	90.41	0.5	(0.29, 0.72)	0.88	(0.82, 0.94)
Rapid	80.83	0.56	(0.43, 0.70)	0.66	(0.55, 0.78)
Pulse force:					
Strong	81.92	0.51	(0.35, 0.66)	0.72	(0.61, 0.82)
Weak	86.14	0.61	(0.47, 0.76)	0.78	(0.69, 0.88)
Pulse shape:					
Slippery pulse	77.84	0.51	(0.38, 0.65)	0.71	(0.63, 0.80)
Fine pulse	73.65	0.37	(0.22, 0.52)	0.67	(0.58, 0.76)
Surging pulse	90.36	0.52	(0.32, 0.72)	0.89	(0.84, 0.95)

CI: confidence interval.

**Table 3:** Agreement between raters in total subjects (diagnosis by visual inspection).

Variables	% Agreement	Kappa (K)	CI of K	AC_1_	CI of AC_1_
Tongue color:					
Pale	78.31	0.54	(0.41, 0.67)	0.72	(0.63, 0.80)
Red	77.84	0.64	(0.54, 0.74)	0.68	(0.59, 0.77)
Fur color:					
White fur	76.64	0.58	(0.46, 0.70)	0.68	(0.59, 0.77)
Yellow fur	79.51	0.57	(0.45, 0.70)	0.73	(0.65, 0.82)
Fur quality:					
Thick fur	83.83	0.54	(0.39, 0.68)	0.80	(0.73, 0.88)
Dry fur	77.24	0.33	(0.16, 0.50)	0.73	(0.64, 0.81)
Special tongue appearance:					
Teeth marked	84.93	0.26	(0.04, 0.48)	0.83	(0.77, 0.90)
Enlarged	84.43	0.41	(0.22, 0.60)	0.82	(0.75, 0.89)
Mirror	95.8	0.76	(0.60, 0.93)	0.95	(0.92, 0.99)

Facial complexion:					
Reddened complexion	84.33	0.70	(0.60, 0.80)	0.79	(0.71, 0.87)
Dark face discoloration	83.83	0.68	(0.57, 0.79)	0.78	(0.71, 0.86)
White complexion	83.13	0.48	(0.32, 0.64)	0.80	(0.73, 0.87)
Pale face and red zygomatic site	87.95	0.58	(0.42, 0.75)	0.86	(0.80, 0.92)
Dark inferior palpebra	84.43	0.47	(0.30, 0.64)	0.82	(0.75, 0.89)

Eye's abnormal condition:					
Congestive eyes	86.82	0.65	(0.52, 0.79)	0.84	(0.77, 0.90)

Body type:					
Underweight	87.42	0.69	(0.57, 0.81)	0.79	(0.70, 0.88)
Overweight	93.41	0.84	(0.75, 0.93)	0.89	(0.82, 0.96)

Tong and mouth:					
Aphtha and tongues sores	92.16	0.55	(0.34, 0.77)	0.91	(0.87, 0.96)
Vigor					
Look powerless and lazy	77.24	0.65	(0.55, 0.75)	0.66	(0.57, 0.76)

CI: confidence interval.

**Table 4:** Agreement between raters in total subjects (diagnosis by listening and smelling).

Variables	% Agreement	Kappa (K)	CI of K	AC_1_	CI of AC_1_
Vocal sound energy:					
Disinclined to speak or speaking at a low volume	76.64	0.61	(0.5, 0.71)	0.67	(0.57, 0.76)
Sputum					
Phlegm rale	90.41	0.74	(0.63, 0.86)	0.88	(0.83, 0.94)
Tongue and mouth:					
Fetid mouth odor	84.93	0.60	(0.47, 0.74)	0.81	(0.74, 0.89)

CI: confidence interval.

**Table 5:** Agreement between raters in total subjects (diagnosis by inquiry).

Variables	% Agreement	Kappa (K)	CI of K	AC_1_	CI of AC_1_
Headache:					
Hot flush in head	89.15	0.74	(0.63, 0.85)	0.86	(0.80, 0.93)
An unpleasant sensation with an urge to vomit	69.87	0.27	(0.12, 0.42)	0.62	(0.53, 0.72)
Tongue and mouth:					
Dry mouth	80.12	0.68	(0.58, 0.78)	0.71	(0.62, 0.80)
Thirst in the mouth	79.51	0.63	(0.52, 0.75)	0.72	(0.63, 0.80)
Temperature:					
Aversion to heat	81.32	0.62	(0.50, 0.73)	0.75	(0.67, 0.84)
Vexing heat in the extremities	90.36	0.46	(0.24, 0.67)	0.94	(0.90, 0.98)
Heat in the palmar and plantar	93.97	0.56	(0.31, 0.81)	0.89	(0.84, 0.95)
Reversal cold of the extremities	90.36	0.64	(0.47, 0.80)	0.89	(0.84, 0.94)
Afternoon tidal fever	91.56	0.52	(0.30, 0.74)	0.91	(0.86, 0.96)
Chest:					
Heat vexation in the chest	87.95	0.76	(0.66, 0.85)	0.84	(0.77, 0.91)
Sleep:					
Vexation and insomnia	81.92	0.63	(0.52, 0.75)	0.76	(0.68, 0.84)
Sweating:					
Night sweating	89.69	0.70	(0.57, 0.83)	0.88	(0.82, 0.93)
Urine:					
Turbid urine	84.82	0.70	(0.59, 0.81)	0.80	(0.72, 0.88)
Vigor:					
Like to lie down	83.13	0.72	(0.62, 0.81)	0.76	(0.68, 0.84)
Feel powerless and lazy	77.1	0.64	(0.54, 0.74)	0.66	(0.57, 0.76)

CI: confidence interval.
